# EEG signal analysis using classification techniques: Logistic regression, artificial neural networks, support vector machines, and convolutional neural networks

**DOI:** 10.1016/j.heliyon.2021.e07258

**Published:** 2021-06-07

**Authors:** Maria Camila Guerrero, Juan Sebastián Parada, Helbert Eduardo Espitia

**Affiliations:** Universidad Distrital Francisco José de Caldas, Bogotá, Colombia

**Keywords:** Data classification, Epilepsy diagnosis, EEG signal analysis, Fourier signal analysis

## Abstract

Epilepsy is a brain abnormality that leads its patients to suffer from seizures, which conditions their behavior and lifestyle. Neurologists use an electroencephalogram (EEG) to diagnose this disease. This test illustrates the signaling behavior of a person's brain, allowing, among other things, the diagnosis of epilepsy. From a visual analysis of these signals, neurologists identify patterns such as peaks or valleys, looking for any indication of brain disorder that leads to the diagnosis of epilepsy in a purely qualitative way. However, by applying a test based on Fourier signal analysis through rapid transformation in the frequency domain, patterns can be quantitatively identified to differentiate patients diagnosed with the disease and others who are not. In this article, an analysis of the EEG signal is performed to extract characteristics in patients already classified as epileptic and non-epileptic, which will be used in the training of models based on classification techniques such as logistic regression, artificial neural networks, support vector machines, and convolutional neural networks. Based on the results obtained with each technique, an analysis is performed to decide which of these behaves better.

In this study traditional classification techniques were implemented that had as data frequency data in the channels with distinctive information of EEG examinations, this was done through a feature extraction obtained with Fourier analysis considering frequency bands. The techniques used for classification were implemented in Python and through a comparison of metrics and performance, it was concluded that the best classification technique to characterize epileptic patients are artificial neural networks with an accuracy of 86%.

## 1 Introduction

According to the World Health Organization [1], there are 50 million patients worldwide suffering from epilepsy, making it one of the most common neurological disorders; when diagnosed in time, it is estimated that 70% of patients could live without seizures or attacks [2]. Many professionals resort to signal spectrum analysis for the diagnosis of epilepsy, in order to identify elements such as peaks and variations in frequency [3]. This can lead to ambiguities in the diagnosis, making it unclear.

Currently, there have been multiple studies and research around the classification of Electroencephalogram (EEG) signals to detect anomalies, epilepsy, sleep disorders, among other special conditions, mostly oriented to the integration with Brain Computer Interface (BCI), a concept that has stood out in recent years [4], a system that reads and interprets the signals directly from an individual, make decisions or execute some instructions from these input data.

In this regard, in [5] are presented several important questions concerning EEG, showing the importance of nonlinear methods in EEG analysis. This work is oriented to determine the applicable technics in the field, like Higuchi's fractal dimension method that is simple and useful.

In addition, to develop a model or a classifier of these signals and to be able to incorporate them into a BCI, it is necessary to understand that the objective of the classification is to be able to use certain metrics to decide the origin of the signals and the factors that affect certain patterns. Choosing the metrics to be used is always the most complex and extensive part of the study problem [6], this information will be called features and its extraction is also a subject of ongoing research. In many particular cases, varied characteristics must be used to make an efficient classification, the most used techniques in the extraction of characteristics are mainly related to time-frequency domain transformations to find the frequency magnitudes in specific parts of the signal, for example the Fourier Transform, and related, as can be seen in [7].

In relation to above, it can be seen in [8] a feature extraction approach based on frequency bands applied to preictal and interictal analysis, i.e., the period before the onset of seizures in the individual and the intermediate period between a series of seizures, respectively. Through the decomposition with Discrete Transformed Wavelet of the signals that are in the Gamma frequency band is made a classification, and then prediction of epilepsy in individuals.

On the other hand, there are classification techniques and proposals aimed at labeling and detecting epilepsy through the characteristics extracted. Machine learning techniques are used to build the classifiers, for example, the case of the author of [9] and [10] who uses the Logistic Regression (LR) because it facilitates the analysis of results in explanatory and predictive terms, since these investigations intend to reduce the dimensions of the signals before applying the logistic regression; likewise, the results show that the classification accuracy is 97.91% with the Gaussian logistic regression model. Artificial Neural Networks (ANN) are present in many studies due to the simplicity of implementation, their advantage to handle large amounts of data and above all, for their ability to learn from examples. In the works [11] and [12], a retro propagation neural network is implemented to classify sharp and acute waves that are known as epileptiform interictal discharges, which are characteristic signs of epilepsy. In addition, a noteworthy work is performed in [13] where a classifier with Support Vector Machines (SVM) is built to detect activity closely related to seizures derived from epilepsy that were recorded in electroencephalograms. This work took into account 5 different types of EEG signals and decomposition was made through the Wavelet transform.

Generally, EEGs are used to determine seizure types and syndromes related to epilepsy, then used for the selection of antiepileptic drugs. Any discovery or distinction in the features of an EEG affects and contributes to the diagnosis, in terms of locating where the disorder or seizures occurs is idiopathic or symptomatic, or specific parts of the epileptic syndrome [14]. Nowadays, it is still necessary to perform detection and visual analysis in the monitoring of the tests, however, the assistance of technologies and frameworks to doctors and professionals in the diagnosis of epilepsy has increased considerably the rate of successful diagnosis [15].

According to [16], in any society, epilepsy is a severe disease given the costs of healthcare and appropriate treatment, therapy, and the sudden convulsion episodes. It is mandatory to settle research processes about integrated and fast neural investigations that helps doctors to efficiently diagnose patients with epilepsy. Commonly, the diagnoses have been defined employing electroencephalograms to determine the brain's electrical activity related to epilepsy.

A noticeably related exploration is seen in [16] about a framework consisting of various feature extraction algorithms (lower threshold, target point selection, and current maxima), energy features, and pattern matching (segment and domain). The authors' model proposal, power, homogeneity, maxima, energy, and physiological traits have been employed. Moreover, the domain matching algorithm has been utilized to identify specific brain regions like lobes, where the convulsion occurs in the previous stage. The authors stated that this model could be employed in a real-time patient monitoring system since this can send a warning message before the convulsion takes place.

The cleaning and pre-processing of the EEG signals are crucial factors, in [17], the time-frequency analysis is used to describe those characteristics that define the signal it observes, the non-stationary signals or those where the frequency changes slowly over time, despite this, it is possible to soften the signals using a Fast Fourier Transform (FFT) and also applying Principal Component Analysis (PCA) to determine the features to feed a Random Forest (RF) model where the experimental results show that it is very accurate in the task of classifying epileptic patients; however, its model is susceptible to signal noise, therefore, a pre-processing is suggested to eliminate the artifacts and noise before using the model.

Regarding other EEG signal applications, patient's age and gender predictions using EEG analysis, there is a type of application belonging to brain-computer interfaces. In this aspect, reference [18] has applied an industrial standard EEG data acquisition device for recording cerebral activities in 60 subjects (male and female) in relaxed position and eyes closed. Deep Bidirectional Long Short Term Memory (BLSTM) network is employed to design a hybrid learning framework to predict age and gender.

In addition, according to [19], human emotions are the result of psychological changes happening in daily activities. In this regard, reference [19] researched emotions identification and analyzed the impact of positive and negative emotions utilizing EEG signals. This paper has studied three types of feelings, namely happiness, anger, and calmness. Using ten subjects, EEG signals are recorded in real-time while watching video clips of different emotions. The features found were classified using Support Vector Machine (SVM) with Radial Basis Function (RBF) kernel to extract such fractal dimension features from raw EEG to detect emotional states.

According to [20], feeling analysis is a fundamental tool to obtain information about emotions from massive data. Sentiment analysis is used to review customers' information and social networking. Thus, since this is useful for e-commerce retailers to advertise products and services based on demographic information, reference [20] examines the impact in age and gender regarding sentiment analyses by gathering information from Facebook users asked about book preferences, age, and gender. Machine Learning (ML) approach is used to analyze feelings, including maximum entropy, Convolutional Neural Network (CNN), Support Vector Machine (SVM), and long short term memory. Various experiments have taken place to determine new observations affecting age and gender for sentiment analyses.

Regarding the use of Fast Fourier Transform (FFT) for EEG signal processing, the work presented in [21] aims at finding the preferred vehicle brand in Malaysia using wireless EEG signals. Four vehicle brand advertisements were used with a video for stimulating subjects' brain signal responses using 14 wireless channels with a headset. From the EEG signal alpha frequency band (8Hz−13Hz) was obtained using Butterworth 4th order filter. The alpha band frequency spectrum is calculated using fast Fourier transform to obtain three statistical features, namely, Spectral Energy (SE), Power Spectral Density (PSD), and Spectral Centroid (SC). The features are useful to build the vector employed in two non-linear classifiers K Nearest Neighbor (KNN) and Probabilistic Neural Network (PNN) to label the subject advertisement.

About Deep Learning (DL), reference [22] presents an analysis of 154 papers published between January 2010 and July 2018 gauging various application domains like epilepsy, sleep, cognitive and affective monitoring, and brain-computer interfacing. Multiple aspects were reviewed like the processing methodology, DL design choices, the results, and possibilities to replicate the experiments. According to analyzes, the EGG amount of data employed across studies fluctuate from less than 10 minutes to thousands of hours; simultaneously, the number of samples observed during the training varies from a few dozens to millions, depending on the extraction of epochs. The authors state that more than 50 percent of studies utilized publicly available data; besides, there has also been a clear move from intra-subject to inter-subject approaches in the last years. Those studies used convolutional neural networks, while others used Recurrent Neural Networks (RNN), mostly of 3-10 layers. Also, almost one-half of the studies trained the models using preprocessed or raw EGG time series; in addition, authors highlighted that frequently studies present poor reproducibility since most of the papers would be difficult, even impossible to reproduce given the unavailability of data and code.

Regarding other works, in [23] is proposed efficient deep and spatiotemporal characteristics for Facial Expression Recognition (FER) based on deep appearance and geometric neural networks. FER has become an essential tool of visual information that could be used to understand human emotions. A three-dimensional (3D) convolution is applied to collect spatial and temporal features simultaneously. Regarding the geometric network, 23 dominant facial markers are used to represent the movement of facial muscle. In this way, it is proposed a fusion classifier to combine the features above mentioned.

In addition to expressions recognition, in [24] is proposed a scheme for FER system based on deep hierarchical learning. In this work, the characteristics extracted from the appearance feature-based network is employed with the geometric elements in a hierarchical structure. The appearance feature-based network acquires holistic characteristics of the face using the preprocessed Local Binary Pattern (LBP) image. In addition, the geometric feature-based network learns the coordinate change of Action Units (AUs) for muscle movement when facial expressions are made. Also, an approach is proposed to generate facial images with neutral emotion using the autoencoder technique. Under this approach, it is extracted the neutral and emotional dynamic facial features without sequence data.

About other CNN related applications, in [25] is shown that the methods combining Correlation Filters (CFs) with the features of a convolutional neural network are suitable for object tracking. In this regard, in [25] is proposed a scale-adaptive object-tracking method. The characteristics are extracted from different layers of “ResNet” to obtain response maps. Then, to locate the target, the response maps are fused based on the “AdaBoost” algorithm. It is proposed an upgrade strategy with occlusion detection for preventing the filters from updating when occlusion occurs.

Other related work is presented in [26], developing a disease recognition model based on leaf image classification using deep convolutional networks. It is presented in a way of training and the methodology used to promote a suitable system implementation in practice. The developed model recognizes different kinds of plant diseases out of healthy leaves and distinguish plant leaves from their surroundings. The authors used “GoogLeNet”, which is considered a robust deep learning architecture to identify types of disease. Transfer learning has been used to fine-tune the pre-trained model.

On the other hand, an appealing approach for the analysis of EEG signal is the use of spectrogram combined with deep learning techniques. In this regard, convolutional neural networks have reached great success in image recognition tasks by automatically learning a hierarchical feature representation. In [27], it is proposed implementing Recurrence Plots (RP) via transforming time-series into 2D texture images allowing the use of deep CNN as classifier. In this way, image representation of time-series introduces different feature types that are not available for 1D signals, and therefore Time-Series Classification (TSC) could be treated as a task of texture image recognition.

About EEG spectrogram application, reference [28] presents the classification of brain-wave response based on low-cost EEG spectrogram to determine how the images are perceived by a person based on their brain waves. The experiments are performed to obtain EEG data based on visual stimuli and convert the data into a spectrogram. For brain-wave response classification based on EEG spectrogram, is used “DenseNet” (deep learning architecture).

Another related application of EEG spectrogram is for diagnosis of the Alzheimer's Disease (AD) at early stages. In this regard, reference [29] shows that it can be addressed with multi-task learning strategy using discriminative convolutional high-order Boltzmann Machine (BM) with hybrid feature maps. This paper describes the implementation of an EEG spectral image classification through “inducing label layer”. For overfitting reduction, the systems are trained with a multi-task learning framework based on identification and verification tasks.

A related application is observed in [30], proposing a two-dimensional (2D) convolutional neural network model to classify Electrocardiogram (ECG) signals into eight classes. The one-dimensional ECG time-series signals are changed into 2D spectrograms through a short-time Fourier transform. In this work, the 2D CNN consists of four convolutional layers for extracting robust features from input spectrograms.

### 1.1 Proposal approach and document organization

In the previous review, it was observed that the most common feature extraction techniques involve Fourier and Wavelet transforms, and statistical metrics that provide outstanding information about behaviors that are not easily identified visually. Besides, it is essential to reduce the dimensions and complexity of these signals given the number of channels classifiers that contrast features and allow labeling of healthy and unhealthy patients. Considering the above aspects, the proposal of this article is to detect characteristics and factors that influence these records to differentiate people with epilepsy or could be susceptible to suffer it through classification models that fit the selected characteristics and provide the highest accuracy indicator.

As mentioned, the fast Fourier transform frequency band technique is common in similar works. However, this research proposed to take two representations for the characteristics extracted from each patient examination; thus, it is performed a band frequency analysis considering healthy and unhealthy patients. In the first place, it was decided to work with the relative power of each frequency band; secondly, with the arithmetic mean of the frequency band values (determined from frequency analysis). The contribution in this paper lies in the band frequency analysis and the measures obtained from these bands. Using this analysis is obtained the input data for the classifiers and the results evaluation to determine the suitable technique for the diagnosis system.

In order to address the problem of epilepsy classification described in previous works, this article presents a quantitative analysis of EEG signals in the frequency domain. Subsequently, to classify patients into epileptic and non-epileptic, different data classification techniques are explored: logistic regression, artificial neural networks, vector support machines, and convolutional neural networks. Each of these techniques is used in the model design to explore alternatives to decide which is the best according to the context of the research.

In each model, 40 sessions were taken corresponding to 40 patients, half epileptic patients and half non-epileptic. It should be remembered that each of these tests is performed according to the 21-channel TCP configuration.

Since the article explores quantitative techniques to differentiate between epileptic and non-epileptic patients, the analysis and results obtained can be applied in the realization of a system to support neurology specialist in diagnosing epilepsy or used in areas where it is not possible to have a specialist.

On the other hand, the monitoring of an epileptic patient may arise as a result of applying the products of this article, since the analysis is made considering previous events of epileptic people from which patterns can be identified to predict whether a person is susceptible to epilepsy.

The organization of the document is as follows, in Section 2, the EEG data used is described, Section 3 details the feature extraction (features used for the epilepsy classification); later, Section 4 describes the experimental design using logistic regression, artificial neural networks, support vector machines, and convolutional neural networks then Section 5 presents the results and the performance metrics used; Section 6 displays the discussion, and finally the conclusions are given.

## 2 EEG data

The technique of encephalography arises from the need to evaluate the dynamic functioning of the brain and its electrical activity. This technique has been used throughout history to evaluate patients with seizures, phenomena and diseases such as epilepsy. The use of electroencephalograms is increasing for preventive diagnosis, as most of these exams have been shown to have distinctive features during epileptic seizures that are represented by electrical discharges in the form of peaks and valleys [31].

For this reason, it is essential for this research to have resources with EEG information and exams that serve as inputs for the construction of classification models and that later can be compared to measure the effectiveness of classification as “Non-epileptic” and “Epileptic”. In this section, an exploratory analysis of the data sets and related information is performed.

### 2.1 Data collection

In the literature review, a well known data source called TUH EEG Corpus has allowed great advances in the field of classification and prediction. This corpus is a product of the work between the Neural Engineering Data Consortium (NEDC) and Temple University Hospital. It makes available a robust set of clinical EEG data covering the period 2002-2013 [32].

The EEGs are presented in an European Data Format (EDF) accompanied by a plain file in which there is a report of the patient, his clinical history and clinical correlation. These tests were collected using “NicoletOne” equipment from NMI. The data were stored in a proprietary format and they do not have the entire original record since it has been pruned by a technician in charge who discards sections of a non-informative nature.

### 2.2 Data description

All these exams use the 10/20 system, each EDF has a set of labels that are not standardized. However, the names of such labels are sufficiently descriptive so their nature and location can be easily related to the 10/20 system and the bipolar assembly. This assembly is traditionally used because it reduces noise and emphasizes the most relevant events, for example peaks in signals. In the case of Temple University Hospital, the most popular mount is the Temporary Central Parasagittal (TCP) mount.

### 2.3 Data selection

**Figure 1:**
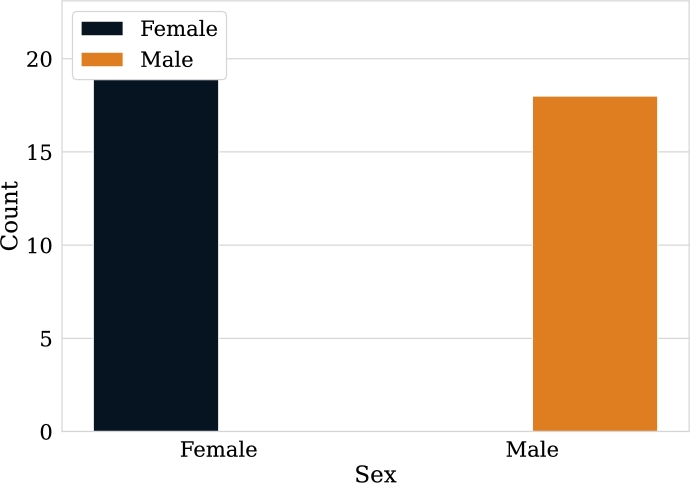
Female and male patient count. Note the predominance of female patients in the dataset.

**Figure 2:**
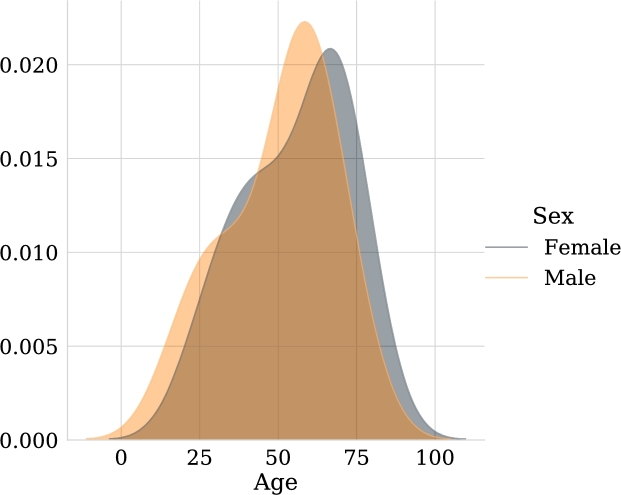
Age distribution of patients. It is notorious that most of the patients are between 1 and 3 years old.

In particular for this research, a small data set was created consisting of 40 different patient examinations (sessions), half of which are patients belonging to the “Non-Epileptic” class and the other half to the “Epileptic” class. Figure 1, Figure 2 show general information about the patients who were selected as part of the dataset.

### 2.4 Channel selection

Each channel in the EDF is classified with a set of labels that are not standardized. However, the names of these tags are descriptive so that their nature and location can be inferred. Even numbers are used to denote electrodes in the right hemisphere and odd numbers refer to those in the left hemisphere.

**Figure 3:**
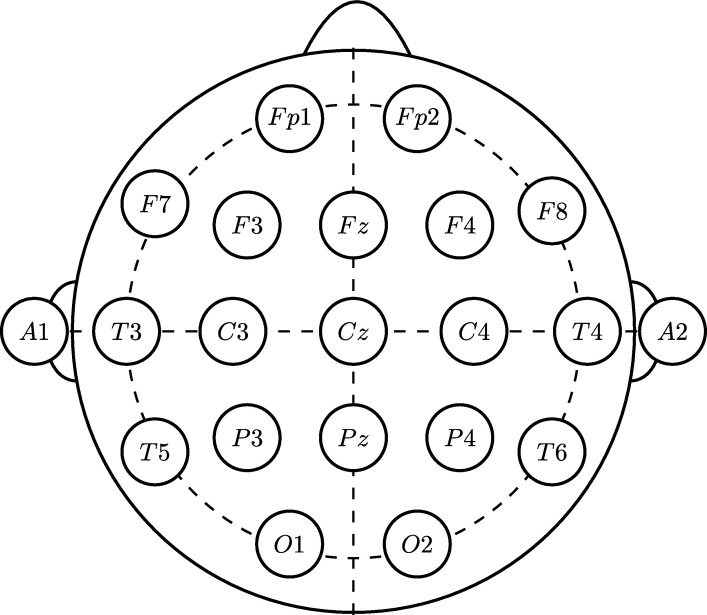
TCP Configuration and channel distribution [32].

The pairs of electrodes are combined to form a bipolar array, which records voltage difference between two electrodes placed in areas of brain activity. This setup is used to reduce noise and emphasize events of interest, such as peaks. Sometimes, this results in a clearer and more easily interpreted signal. However, there are combinations of electrodes that are much more vulnerable to specific artifacts. Several assemblies are used at Temple University Hospital [32], although one of the most popular bipolar assemblies among neurologists is the Temporal Central Parasagittal (TCP) or also known as the double-banana assembly. Therefore, for the extraction of characteristics it is worked with all 21 channels of the bipolar assembly as shown in Fig. 3.

## 3 Feature extraction

In order to extract features from the data presented in the previous section, a spectral analysis is performed on these exams. This analysis seeks to quantify the oscillatory activity of the signals at different frequencies of the signal [33]. This method is considered one of the main and accepted methods in the field of neuroscience. However, the success of the feature extraction depends on the approach to this spectral analysis, as there are no officially standardized methods that prevent inconsistent results.

The selected approach for this research was decomposition into frequency bands. This technique resorts to the application of Fourier Transform to the entire signal by means of Fast Fourier Transform which facilitates the extraction of relevant events (patterns, peaks, valleys, among others). From this transformation from time domain to frequency domain and the obtention of such events, it is possible to identify and label epileptic and non-epileptic patients.

**Figure 4:**
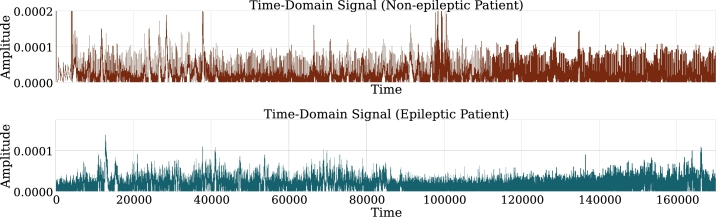
Comparison between the signal of an epileptic patient and a non-epileptic patient in time domain. Crucial differences in the two signals cannot be distinguished given the nature of the signal.

As an example, the signals in the time domain for patients of both classes can be seen in Fig. 4. The signals exhibit behavior from which little information can be extracted, if the patients were not labeled, the distinction would not be evident.

**Figure 5:**
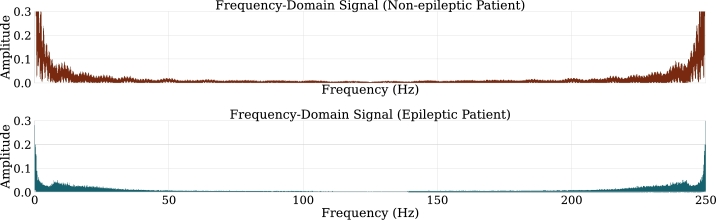
Comparison between the signal of an epileptic patient and a non-epileptic patient in frequency domain. Through this transformation, the difference between the components of each signal can be noted.

However, by passing such signals into the frequency domain, certain differences between patients can be identified. It is visualized in Fig. 5 that the epileptic patient has low frequency dominant activity, this is probably due to severe damage to the brain [7].

The process of transforming time domain to the frequency domain is:

1. Taking the EEG signal in the time domain.
2. Apply the Fourier transform via FFT.
3. Determine the frequency bands taking into account the frequency spectrum of the EEG signal (obtained from the FFT).
4. Sampling the data (frequency bands).
5. Calculate the absolute and average values.

To identify the different features involved in the classification system it is performed an exploratory analysis to observe the behavior of the signals and to detail the frequency bands. Below, an example of two EEG exams belonging to both an epileptic and a non-epileptic patients to illustrate the process followed to obtain the features (frequency bands) of each channel.

After establishing the classification features the different systems used were validated with 40 exams belonging to the subset that was made of the corpus [32]. In the following sections, the process followed by each EEG examination is illustrated, which allowed the extraction of information from the frequency domain for each patient analyzed.

### 3.1 Non-epileptic patient

**Figure 6:**
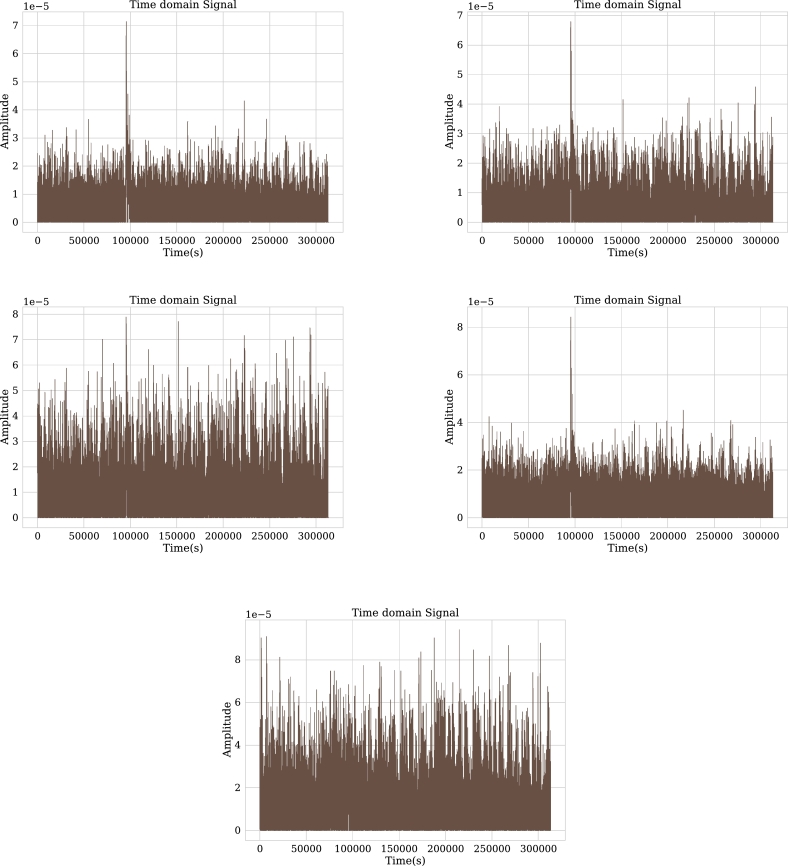
Channels F3, F7, C3, P3, and T4 in time domain.

The patient considered as a demonstrative example is a 75 year-old man; his exam is performed due to changes in his mental state. Upon arrival at the clinic, he is intubated and given a sedative called Midazolam; normally, the effect of this sedative is quickly stopped. However, the patient does not wake up; for this reason, the exam is performed during his state of drowsiness. The selected channels, according to a visual analysis of this exam are: F3, F7, C3, P3, and T4. Fig. 6 shows these channels in time domain.

#### 3.1.1 Channels F3, C3 and P3

The relative power information for these channels is tabulated in Table 1. The band with the highest value is the Delta band. There is a correspondence between the description of the exam clinic correlation and the results of the table given the deep sleep state thanks to the effects of the sedative, the Delta bands are the ones that abound in the signal.

**Table 1:** Summary of relative power for each channel classified by frequency bands.

Channel	Alpha	Beta	Delta	Gamma	Theta
F7	0.0069	0.0062	0.9561	0.0174	0.0131
T4	0.0197	0.0163	0.9187	0.0186	0.0264
F3	0.0172	0.0132	0.9113	0.0306	0.0274
C3	0.0185	0.0153	0.8738	0.0632	0.0287
P3	0.0241	0.0183	0.7439	0.1764	0.0367

#### 3.1.2 Channel T4

In this channel there are 91.8% of frequencies corresponding to the Delta band and 2.6% of the Theta band, the rest of the frequency bands have very small values respect to these bands (Delta and Theta).

#### 3.1.3 Channel F7

**Figure 7:**
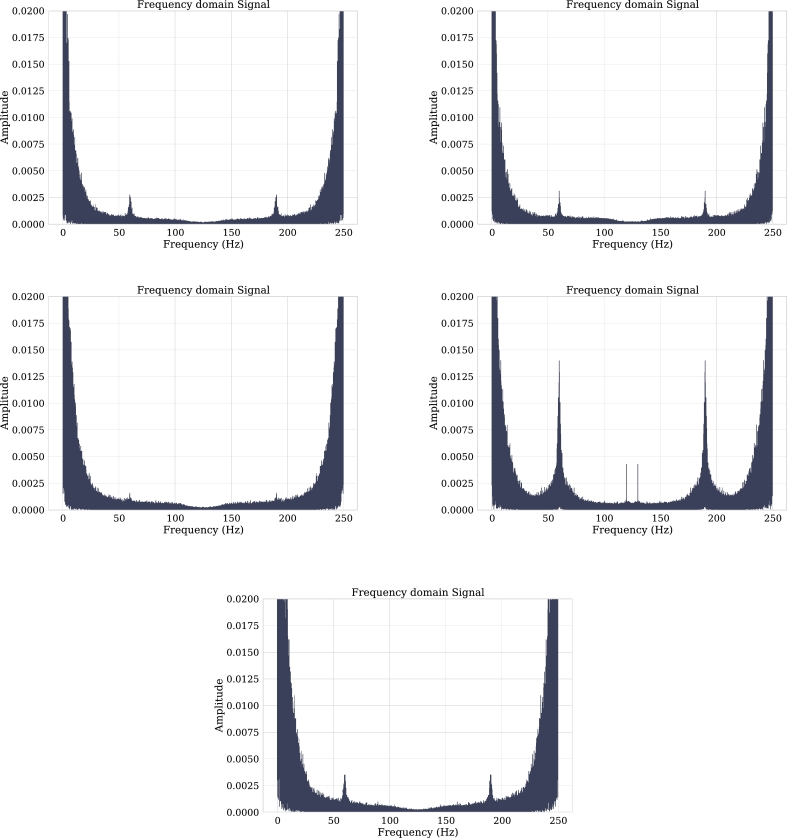
Channels F3, F7, C3, P3 and T4 in frequency domain.

In the time domain it is observed that the signal ranges from 50 to -50 microvolts. By moving such a signal into the frequency domain it can be seen in Fig. 7 that its amplitude range is between 0 and 0.05 microvolts and it has 0.15 microvolt amplitude peaks. When extracting the characteristics shown in Table 1, it can be seen that 95.6% of the signal is contained in the Delta band.

### 3.2 Epileptic patient

**Figure 8:**
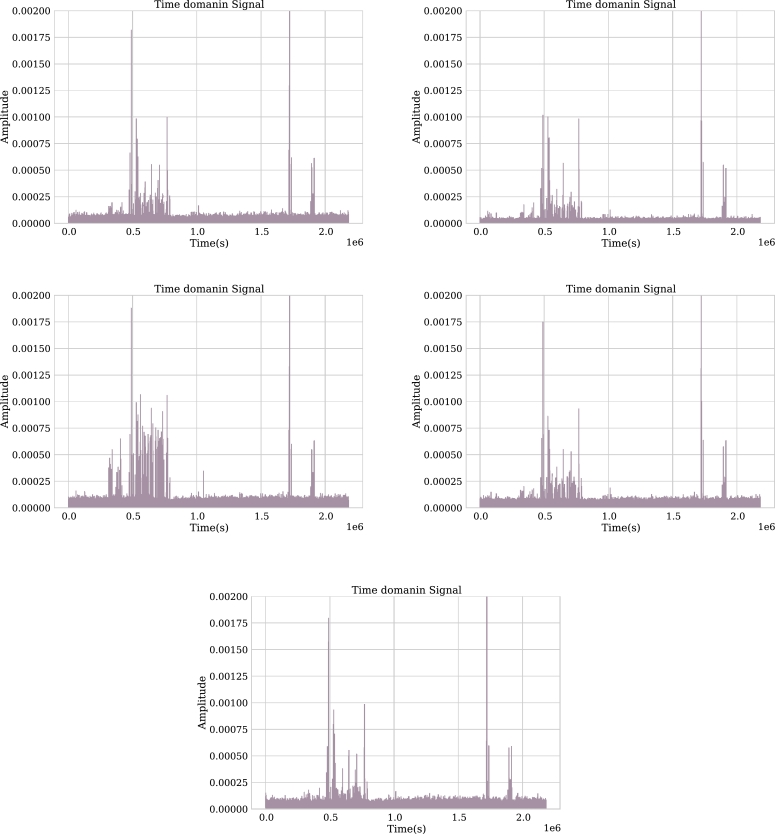
Channels F3, F7, C4, P4 and T5 in time domain.

Now, as an example for an epileptic patient, the exam of a 75-year-old woman with urinary incontinence problems is discussed. The exams were performed on the patient in an unconscious state. It should be noted that this woman has been previously treated with anticonvulsants such as Dilantin and Lorazepam. The channels next analyzed are F7, C4, T5, F3, and P4 (Fig. 8).

#### 3.2.1 Channels F7, C4 and T5

**Figure 9:**
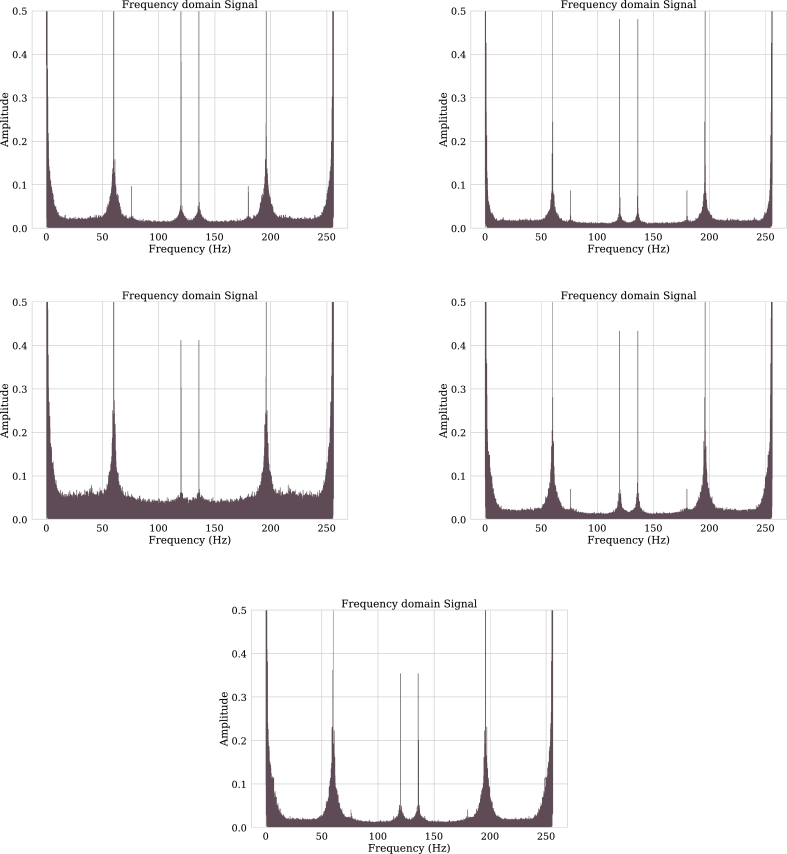
Channels F3, F7, C4, P4 and T5 in frequency domain.

Channel F7 is part of the frontal lobe of the brain, channel C4 is identified as inner right medial, and channel T5 as outer left rear. As can be seen in Fig. 9, the channels have low activity in the frequency domain with a high presence of the Delta band (0-4 Hz). According to [34] this may be related to brain injury.

In Table 2, the relative power information for these 3 channels is given, where it is noticeable that the delta band predominates and as previously mentioned, the high presence of this band generally indicates brain injuries.

**Table 2:** Summary of relative power for each channel classified by frequency bands.

Channel	Alpha	Beta	Delta	Gamma	Theta
F7	0.0149	0.0484	0.4481	0.2237	0.0527
C4	0.0279	0.0320	0.4599	0.0778	0.0872
T5	0.0209	0.0182	0.5179	0.0451	0.0367
F3	0.0167	0.0379	0.4415	0.1481	0.0636
P4	0.0253	0.0185	0.5307	0.0270	0.0904

#### 3.2.2 Channel F3

Fig. 9 shows that the amplitude of this channel is low and, as in channels F7, C4, and T5, the predominant band is Delta. It can also be noticed in Table 2 that the Gamma band occupies almost a third of the Delta band. From this information, it can be concluded that the patient has problems related to abstract thinking and memory.

#### 3.2.3 Channel P4

This channel of the parietal lobe, which is located in the right rear part of the brain, has a high presence of the Delta band for this patient. Table 2 shows that more than half of the frequency components are contained in this band, reflecting damage to the back of the brain.

## 4 Experimental design

Within the state of the art review for the elaboration of this article, several approaches were found to carry out the extraction of features and the classification of patients according to these features. To achieve the results obtained in this article, several experiments were carried out combining the representation of the data (relative and average potential) with each one of the classification techniques.

It is this combination between representations and classification techniques together with an exhaustive experimentation that allows finding the best results according to the context and conditions of the data. For each of the techniques applied, this article describes the two best experiments obtained with each data representation, therefore, there are four models described for each classification technique.

### 4.1 Description

Within the research carried out, several experiments took place to observe which configurations show changes in the performance metrics and, in this way, modify, remove or add values to parameters looking for improvement for these.

These performance metrics are obtained from the results listed in the confusion matrix. In each of the four quadrants of this matrix lie the corresponding sample quantities according to the predictions made by each model. Each of them is defined as:

• True negatives: those samples whose class is zero and, whose model managed to classify as such.
• False positives: those samples whose class is zero, but whose model is classified as 1.
• False negatives: in this case, the model classified the class 1 samples as class 0 samples.
• True positives: in this space is found the number of tests that are class 1 and that the model classified as such.

These values are positioned in a 2×2 matrix as shown in Table 3. From the values found in the confusion matrix, the following three metrics are calculated.

• Accuracy: this is the fraction of predictions that the model made correctly [35].
  (1) $$\text{ Accuracy }=\frac{TP+TN}{TP+TN+FP+FN}$$
• Precision: This metric determines the proportion of correctly classified positive identifications [35].
  (2) $$\text{ Precision }=\frac{TP}{TP+FP}$$
• Recall: this is defined as the proportion of positives that were correctly identified [35].
  (3) $$\text{ Recall }=\frac{TP}{TP+FN}$$

**Table 3:** Position of samples on confusion matrix.

	Predicted Values	
Actual Values	True Negatives	False Positives
	False Negatives	True Positives

### 4.2 Logistic regression

For the first group of experiments with classification techniques, logistic regression was used. This technique provides the mechanism for linear regression to classification problems. The result of the classification is actually a value between [0,1], which is interpreted as the probability h(x) that the class of *x* is 1. In particular, the sigmoid function used in the logistic regression is the logistic function, defined as:

(4) $$f(z)=\frac{1}{1+e^{-z}}$$

where *z* is of the form: $z=\beta_{0}+\beta_{1}x_{1}+\beta_{2}x_{2}+...+\beta_{n}x_{n}$, where $x_{1}$ to $x_{n}$ represent the values of the *n* attributes and *β* or to $\beta_{n}$ represent the weights [36].

As mentioned throughout this article, different configurations were tested in hopes of changing and improving the metrics of each model. For this reason, additional parameters to those explained above were applied to the logistic regression models. The first of these was the *C* coefficient called the Regularization Inverse. This is a control variable that softens and regulates the overfitting of the model [37]. On the other hand, there are the penalties or regularizations to reduce the variance of the model and therefore avoid overfitting [38].

#### 4.2.1 Relative power of frequency bands

The first experiments with logistic regression were performed with the input data represented in the relative power of the bands. Although only two experiments are listed in this section, these are the result of comparing 6 previously developed models, in which the best results were not observed by applying the total of the samples chosen for this research.

**Figure 10:**
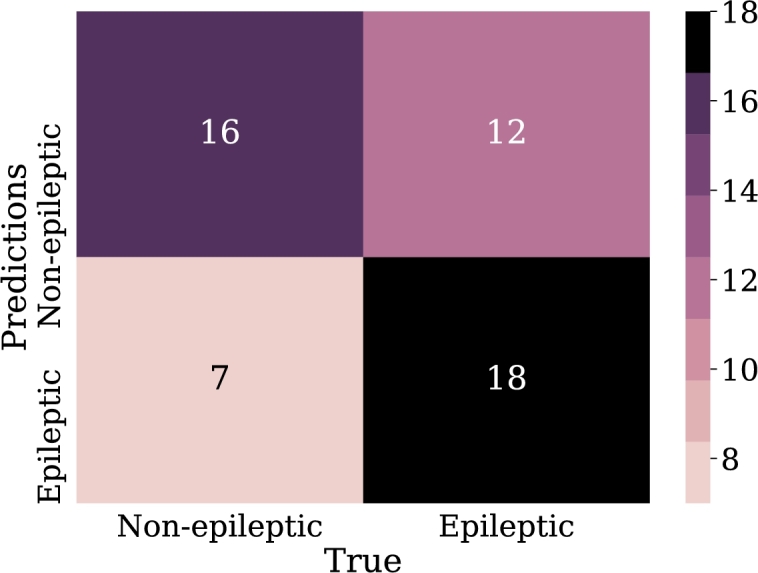
Confusion matrix Model LOG-REG-01. From left to right and from top to bottom the number of true negatives, false positives, false negatives, and true positives.
• LOG-REG-02:
  Based on the analysis and the results obtained in the previous configuration, for this new model, a new strategy was introduced in order to avoid underfitting. For this reason, three new characteristics were added to the input data and therefore, there are now eight input characteristics in the model; these new features are:
  **–** Product of the alpha and gamma band values.
  **–** Product of beta and delta band values.
  **–** Average of the percentage coefficients of the 5 bands.
  It should be remembered that this is done to avoid underfitting the model, which may be caused since current models have fewer samples for training. Finally, “liblinear” was applied as an optimization algorithm, which is a linear classifier. It uses a coordinate descending algorithm that solves optimization problems by successively performing rough minimization along coordinate directions or coordinate hyperplanes [40].
  Since, the algorithm performs a classification one versus the rest, this may represent disadvantages in speed issues with respect to “lbfgs”, however, as [40] noted, linear classification presents outstanding, results in training for large and scattered data with a large number of instances and features wanted. These aspects are considered to perform the application using this technique.
  This model obtained an accuracy of 73% and an recall of 68%. Although these values are not much better than the previous model, and in fact the value of recall was lower, the precision was considerably increased to 74%, thus increasing the number of correctly classified epileptic patient samples (Fig. 11).

• LOG-REG-01:
  Since previous experiments, the best results were not achieved using the total amount of samples selected in this research. In this model, only 210 samples were taken, 105 were from epileptic patients and the other 105 from non-epileptic patients. This, in order to avoid problems of underfitting.
  The input features for this model are the 5 EEG frequency bands (alpha, beta, gamma, theta, delta). These bands are expressed in percentage according to their presence in the signal. The optimization algorithm of the problem is “lbfgs”, which is analogous to Newton's method; however, it has a particularity that consists of the use of an estimation of the inverse of the Hessian [39]. The application of this algorithm reduces memory usage, allowing for a minimum of the fastest function to be found.
  As a penalty, L2 was applied. It should be remembered that regularization allows for a reduction in the complexity of the model, which is also part of the strategies to avoid overfitting. In this case, the weights are penalized in proportion to the sum of the squares of the weights. The L2 regularization helps to bring the weights of outliers, i.e. those distant samples of the epileptic and non-epileptic classes, to values close to zero.
  From the data of the confusion matrix in Fig. 10, the performance metrics for this model are calculated. In this case, 72% was obtained for recall metric and 70% for accuracy metric which may indicate acceptable model performance. However, the precision of the model was only 60%, which means that just over half of the epileptic patient samples were correctly predicted by the model.

**Figure 11:**
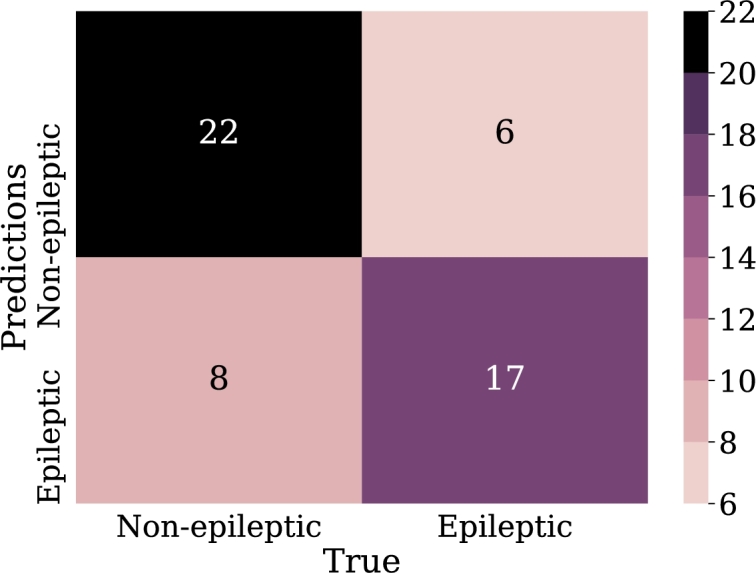
Confusion matrix Model LOG-REG-02. From left to right and from top to bottom the number of true negatives, false positives, false negatives and true positives.

#### 4.2.2 Arithmetic mean of frequency bands

**Figure 12:**
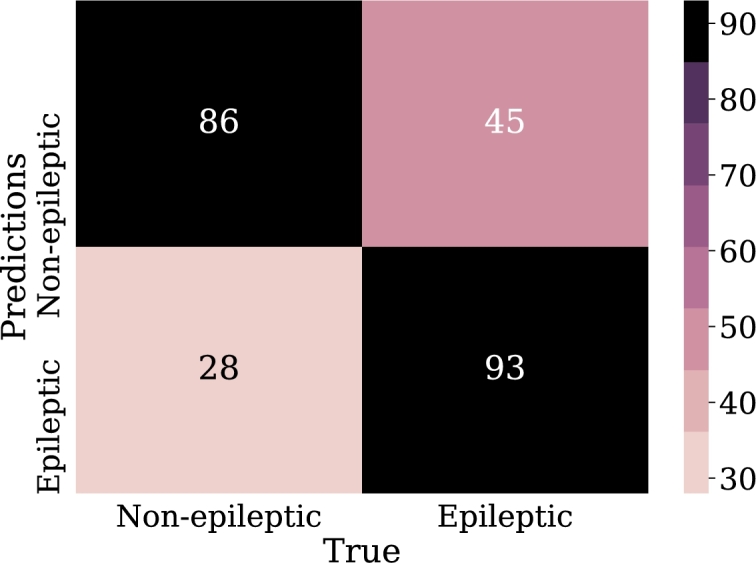
Confusion matrix Model LOG-REG-03. From left to right and from top to bottom the number of true negatives, false positives, false negatives, and true positives.
• LOG-REG-04:
  Based on the improvements observed in model LOG-REG-03, in this new model two new features were added which were generated from the average and median values of the frequency bands; therefore, this model has 10 features in the input data. As in the previous model, discretization was applied to the data, which further increases the dimension of the input data, since it is now discretized based on 10 features.
  Despite having generated new characteristics counting on obtaining improvements in the metrics again, these decreased with respect to the previous model. The accuracy was 66% and the precision 63%. On the other hand, the recall was 70%. These values were extracted based on values in Fig. 13. The values of this model are lower compared to LOG-REG-03 model, thus, there may be overfitting compared to the mentioned model.

• LOG-REG-03:
  Unlike the previous models, in this one the total samples were applied, and therefore, there are 588 training samples and the remaining 252 for the validation. This model was preceded by other two in which this same amount of data was used. However, they did not show satisfactory results, that is why, as in model LOG-REG-02 (Fig. 11), new characteristics were generated hoping to obtain the same improvements observed for that model. These new features were:
  **–** Product of beta and gamma band values.
  **–** Product of delta and gamma band values.
  **–** Product of the beta and theta band values.
  In addition, the input data was pre-processed through discretization via the KBins algorithm. With this it is possible to generate small groups of samples with similar characteristics and classified as epileptic or non-epileptic. Now the characteristics are divided into discrete values [41].
  Continuing with the approach used in the previous section, for these new models an exploration of the optimization algorithms was made, that is why the “lbfgs” algorithm was applied again. On the other hand, the discretization of the input data increases the number of characteristics of these. Thus, the “lbfgs” algorithm can represent improvements since the dimension of the data grew [42].
  As in the last logistic regression model, new features were added from polynomial combinations of the existing features. The coefficient C=10 was taken, using “lbfgs” as the optimization algorithm and ridge 2 as the penalty. From the data of the confusion matrix in Fig. 12, it is obtained an accuracy of 71% and precision of 67% while recall was 77%. In comparison with previous models, there is an increase in the metrics in general. It should be noted that in this experiment the number of samples was increased, which may make the model more susceptible to overfitting problems than previous models.

**Figure 13:**
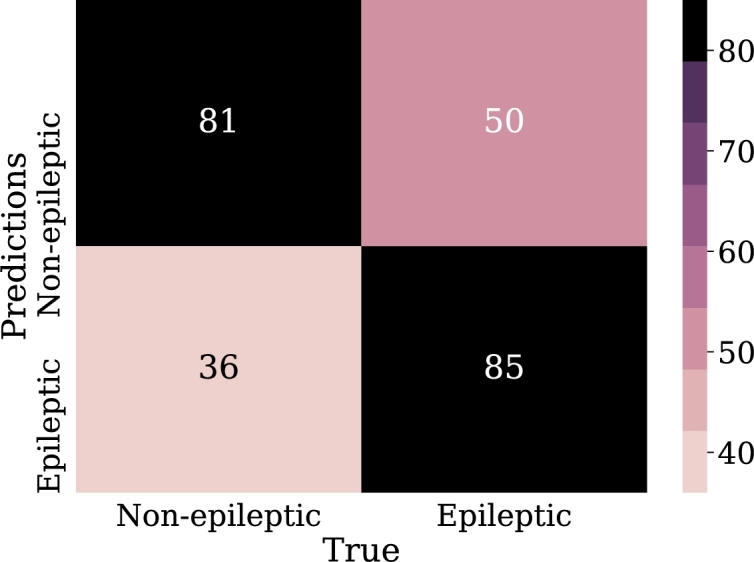
Confusion matrix Model LOG-REG-04. From left to right and from top to bottom the number of true negatives, false positives, false negatives, and true positives.

### 4.3 Artificial neural networks

The second technique to be evaluated is the artificial neural network technique. A neural network is nothing more than an architecture of interconnected nodes. The artificial neural network stores and processes data through the connections that exist between its nodes generated by a learning process that distinguishes patterns in the training data [43].

(5) $$a=\phi \left(\sum_{j}w_{j}x_{j}+b\right)$$

Where $x_{j}$ are the unit inputs, $w_{j}$ the weights, *b* the bias, *ϕ* is the non linear activation function y *a* is the activation unit.

The experiments are performed by taking two different representations of the frequency bands (relative power and arithmetic mean). The following subsections present the experiments performed by altering parameters between them to obtain efficiency in the classification of epileptic and non-epileptic patients.

Feed-forward neural network are characterized by their relationships in degree without cycles and sequential. The multilayer neural network was the subtype selected for these experiments. Each node in one layer is connected to a node in the next layer. There are input layers (first layer), hidden or intermediate layers, and an output layer (the last layer of the neural network). In the case of this investigation, there is only one node in the last layer as it is a binary type classification.

#### 4.3.1 Relative power of frequency bands

**Figure 14:**
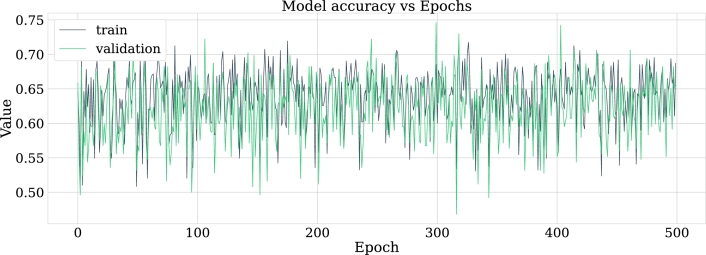
Accuracy of the NN-FF-03 model for training and validation data in 500 epochs.
  With this configuration a score of 78% of the model was achieved, for the classification of the patients an accuracy of 86.1% was obtained. These results are high and indicate that the number of hidden layers has a positive impact on the metrics considered. The confusion matrix in Fig. 15 clearly illustrates the classification of the test samples.

• NN-FF-01:
  This experiment has an architecture for the neural network described next. It has an input layer that receives five features (one for each frequency band). It has six hidden layers with 25, 10, 14, 15, 20, and 10 nodes respectively. The optimization method employed is the *LBFGS*, which commonly converges faster than other methods. The memory-limited LBFGS or BFGS algorithm belongs to the quasi-Newton family of methods that work similarly to the Broyden-Fletcher-Goldfarb-Shanno (BFGS) algorithm using a limited amount of computer memory. It became popular for parameter estimation in machine learning problems. Its main purpose is to minimize the unrestricted values of the real value where there is a differentiable scalar function [44]. The penalty parameter L2 is α=0.00001 and the trigger function used is “ReLU”.
  At the time of running this experiment it was necessary to adjust the number of iterations to 3500 for the optimization function to converge. Similar to how it was done in the logistic regression subsection, it was important to scale the features before training the model, tests were done and for this experiment the standard scaler obtained better results than the robust scaler. Fig. 14 shows the behavior of the model with respect to accuracy metrics.

**Figure 15:**
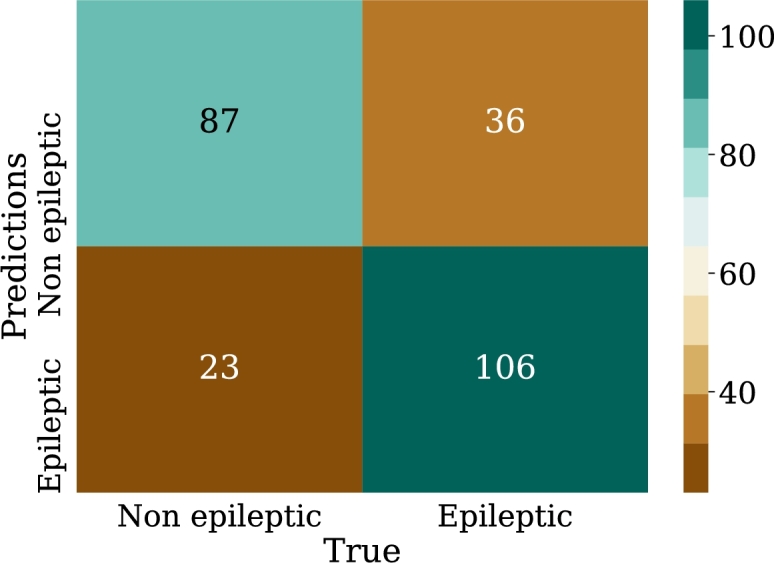
Confusion matrix of model NN-FF-01. From left to right and up to down the number of true negatives, false positives, false negatives and, true positives.
• NN-FF-02:
  Taking the NN-FF-01 experiment as a starting point, the layers described below begin to be configured:
  **–** Layer 1 (Input Layer): This layer has five dimensions, the ReLU activation function is applied to the point product between each dimension and a matrix of weights that is initialized by the same layer; therefore the output of this layer is the product between the characteristics and those weights.
  **–** Hidden layers 2 and 4: A dropout technique is applied taking into account that being a small data set the model may present an over-adjustment. In summary, this technique randomly sets input nodes at 0 with a certain frequency during the time it takes to train the model, the rest of the nodes that are not at 0 are expanded by a value corresponding to $\frac{1}{1-\mathrm{rate}}$ so as not to affect later the sum of all the input nodes.
  **–** Layer 3: This is similar to the input layer where a ReLU activation function is applied.
  **–** Layer 5 (Output Layer): Sigmoid function that transforms the product between the inputs and the weights.
  Standard feature scaling was performed in 700 epochs with the Adam optimization method. The loss calculation was performed with the help of the binary cross entropy function, this is an iterative method in which first a random set of values are generated and then the parameters are updated to generate better values, more approximate in the sense of Kullback-Leibler, in the next iteration. The advantage of this method is that it can be applied both to deterministic problems and to problems with noise [45], the results for the model loss are 3.44% and the overall model score is 86.1%. The performance of the experiment is shown in Fig. 16.

**Figure 16:**
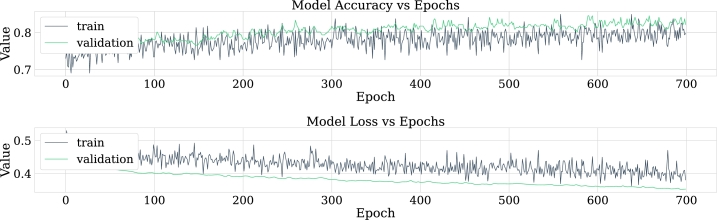
Accuracy and loss of NN-FF-04 model for training and validation data in 700 epochs.

#### 4.3.2 Arithmetic mean of frequency bands

**Figure 17:**
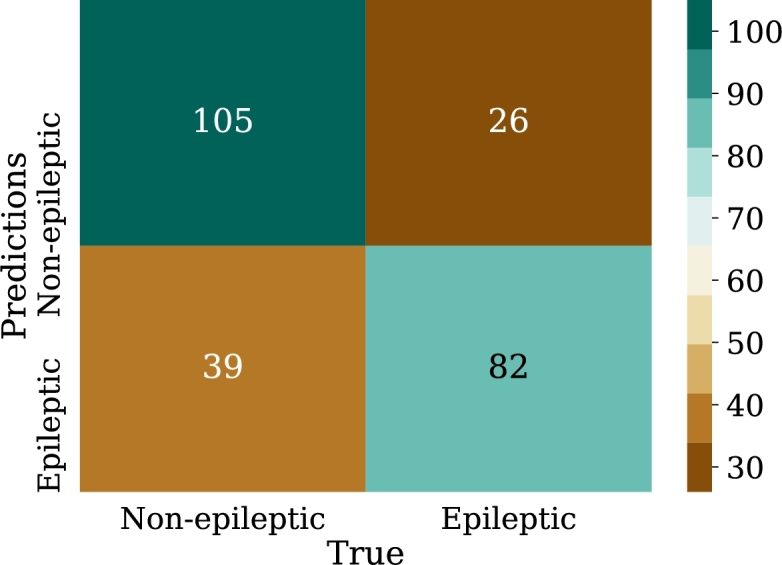
Confusion matrix of model NN-FF-03. From left to right and up to down the number of true negatives, false positives, false negatives, and true positives.
• NN-FF-04:
  As in the NN-FF-03 experiment, the use of the “ReLu” activation function has worked better than the Hyperbolic Tangent function. The setup for this experiment differs in an increase in the number of layer neurons. Thus, the first hidden layer had 128 neurons, the second layer consisted of 64 units, the third layer had 32, the fourth and fifth layers had 16 and 8 neurons respectively. It is observed that there is an improvement in the metrics and the accuracy of the model is highlighted as it reaches 81% when classifying the test data.
  Such results can be better evidenced through the confusion matrix in Fig. 18, the increase of the values in the upper-left and lower-right corners, since there is a count of the predicted values against the real values of the training data set. The increase in these corners indicates a marked improvement in prediction for the test data set. At the same time, Fig. 19 shows the loss curve for the validation data, which is close to 15%, an indicator that is remarkable since it suggests that the model has performed well in comparison with other models.

• NN-FF-03:
  In this experiment it was used the “RMSProp” algorithm, similar to the gradient descent algorithm, with the difference that it restricts oscillations in the vertical direction of the gradient optimization, thus reaching faster the minimum of the function, “RMSProp” addresses and adjusts the problem of excessive accumulation of the gradient along the iterations, multiplying the previous accumulation by a parameter *ρ* that will make its weight to reduce along the iterations in an exponential way. Thus, the learning rate will not be so drastically reduced [46]. The configuration for this model was 32 neurons in the first hidden layer, 64 in the second layer and 16 in the last hidden layer. These layers have the function of “ReLu” activation.
  The results for this experiment are 74% for classification accuracy, other metrics can be calculated from the confusion matrix shown in Fig. 17.

**Figure 18:**
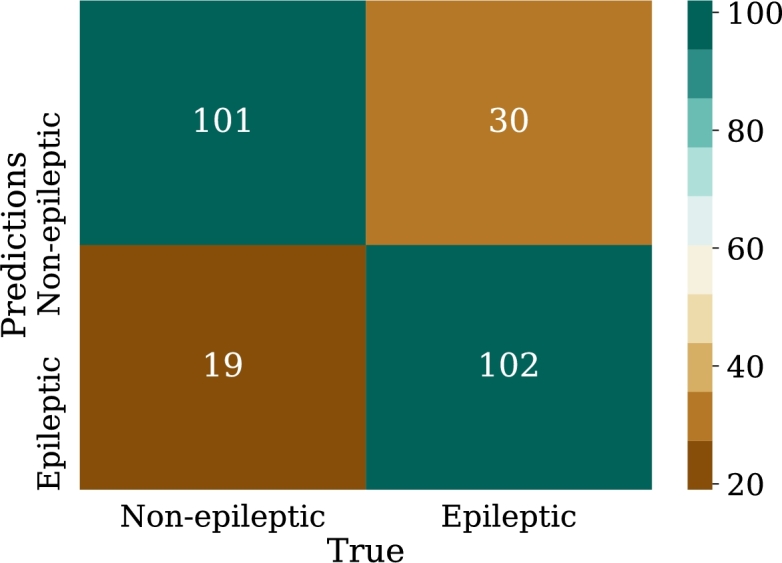
Confusion matrix of model NN-FF-04. From left to right and up to down the number of true negatives, false positives, false negatives, and true positives.

**Figure 19:**
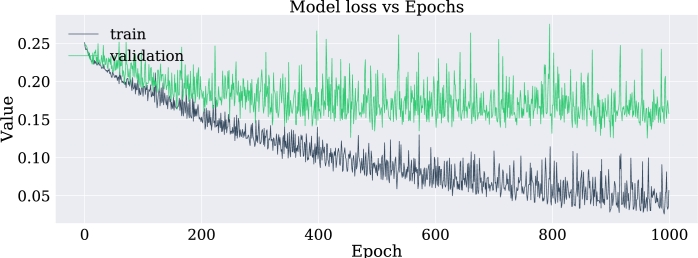
Loss curve of the NN-FF-04 model for training and validation data in 1000 epochs.
  In this case, an overfitting effect has been observed which according to [22] may be associated with the little amount of data available to carry out the training.

### 4.4 Support vector machines

In order to improve the results obtained in the previous experiments, this section presents experiments carried out with support vector machines, as a technique for classifying examinations. Support vector machines are linear algorithm applied in classification, regression, density estimation, etc. In the simplest case of binary classification, like in this paper, SVM find a hyperplane that separates the two classes of data with as wide a margin as possible [47]. The models made with this classification technique are described below.

#### 4.4.1 Relative power of frequency bands

For this group of experiments it is used as kernel the radial base function as well as a pre-processing of the input data through scaling techniques which will be described for each experiment.

**Figure 20:**
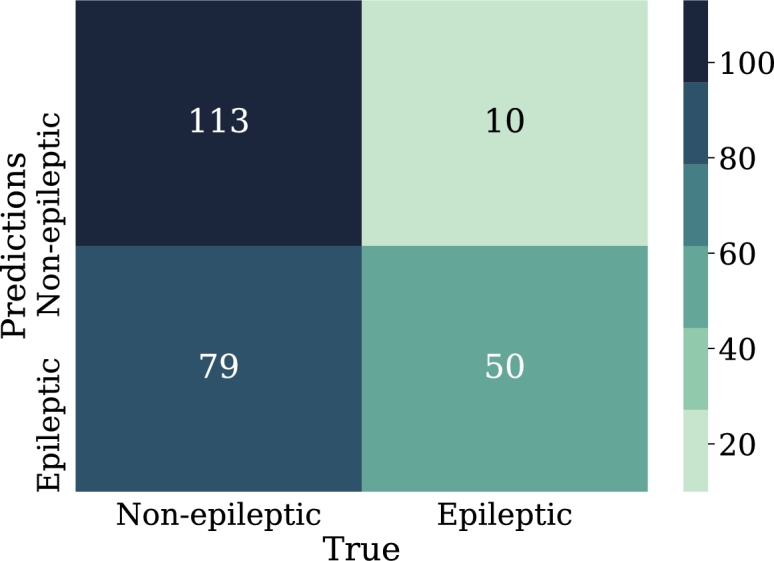
Confusion matrix of model SVM-01. From left to right and from top to bottom the number of true negatives, false positives, false negatives, and true positives.
• SVM-02:
  In this new model, a standard scaler for input data normalization was implemented, which allows the data variance to be equal to 1. This data pre-processing has an outstanding behavior when applied together with the radial-based kernel, as mentioned in [50]. As seen, the radial-based kernel separates the data according to centroids. Therefore, it is even easier for the kernel to generate these centroids. The input data is now within the same range. Equations (6) and (7) describe the behavior of the kernel for this experiment.
  Compared to the previous model, quite similar results were obtained, for example, the recall gave a result of 39%, in addition, the accuracy and precision values were 64% and 79% respectively. These results are reflected in the low numbers of samples in the upper right and lower right corners, in the confusion matrix in 21, whose values are lower than their peers in the left column. This indicates a low success rate in predicting epileptic patients.

• SVM-01:
  Percentile-based scaling was used for this experiment. In order to separate the data according to their class (epileptic and non-epileptic), this scaler is applied, which removes the median and scales the data according to the quantile range (default to *IQR*: interquartile range). The *IQR* is the range between the first quartile (25th quartile) and the 3rd quartile (75th quartile) [48]. Along with this pre-processing, the radial base function was applied as a kernel. This combination of scaler and kernel applications allows the separation of the samples. The radial base function processes the data generating new characteristics from the distance between points, and thus finding centroids. Equation (6) describes this.
  (6) $$K(x,x_{0})=e^{-\gamma ||x-x_{0}|{|}^{2}}$$
  Where *γ* controls the influence of centroids. These centroids define boundaries, so the higher the value of *γ*, the less influence these centroids have on the decision boundary and therefore the decision boundary is less extensive [49]. The value of *γ* (gamma) for this model is given according to Equation (7).
  (7) $$\gamma =\frac{1}{m\times S^{2}}$$
  Where *m* is the number of characteristics, for this case m=5 and $S^{2}$ is the variance of data, which, as mentioned above, is affected by the application of escalation. In this case, the value of the variance is $S^{2}=0.976$, therefore the value of *γ* is 0.204.
  For this model, a percentage of 64% was obtained for accuracy. The precision was 80%, but the recall metric does not give a suitable result, as it is only 39%. Fig. 20 shows the results of the confusion matrix for this experiment.

**Figure 21:**
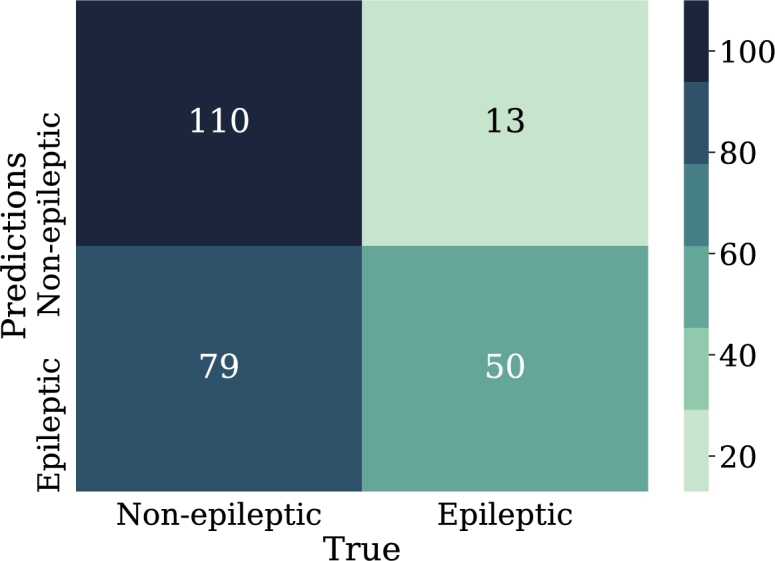
Confusion matrix of model SVM-02. From left to right and from top to bottom the number of true negatives, false positives, false negatives, and true positives.

#### 4.4.2 Arithmetic mean of frequency bands

**Figure 22:**
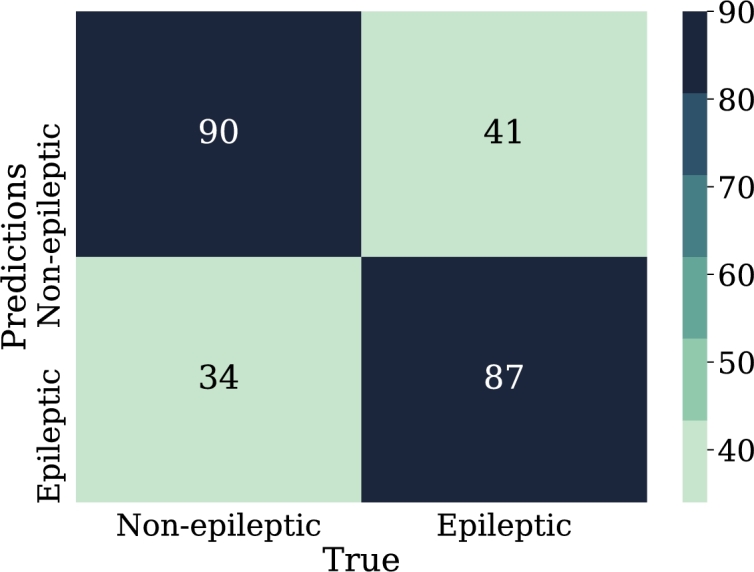
Confusion matrix of model SVM-03. From left to right and from top to bottom the number of true negatives, false positives, false negatives, and true positives.
• SVM-04:
  As said so far, the models described here are the result of testing various configurations. Based on the results obtained, in this model was applied preprocessing of the data applying scaling by absolute maximum, which scales and translates each characteristic so that the maximum absolute value of each is 1 [52].
  As seen, the radial basis kernel works remarkably when the data have been pre-processed. That is why this kernel was applied in this model. In this case, the value of the parameter *C* is 10000. It should be remembered that this value is the inverse of the regularization value, so even though the regularization is small, applying this value represented an improvement in metrics compared to models made using support vector machines.
  This setup allows to get an accuracy of 77%, precision of 78% and recall of 74%. Fig. 23 shows that this model has obtained so far the best results of the experiments made with support vector machines, given the amount of samples in the upper-left and lower-right corners of the confusion matrix.

• SVM-03:
  In this experiment a new kernel is applied, in this case a polynomial one, which adds new features from polynomial combinations of all existing features. Equation (8) defines the behavior of this kernel.
  (8) $$K(x,x^{\prime })={\langle x,x^{\prime }\rangle }^{d}$$
  where *x* and $x^{\prime }$ represent input features for the model, in this case, frequency band values, and *d* is the polynomial degree.
  For this model, the same scaling applied in the SVM-01 model was used. On the other hand, for this model the Equation (9) defines gamma value.
  (9) $$\gamma =\frac{1}{m}$$
  In this case, this kernel was applied to observe how it behaves with respect to the radial-based kernel. Since, as suggested in [51], this kernel allows to generate non-linear borders, that allows to separate samples with similar features that are spatially close, but are of different kind.
  For this model, the kernel with a grade 5 polynomial was implemented. To reach this degree of polynomial, experiments were conducted with higher and lower values where the kernel with the best performance was grade 5. As can be seen in Fig. 22, the confusion matrix, from which the metrics are calculated, which in this case were 70% for accuracy, 68% for precision and 72% for recall.

**Figure 23:**
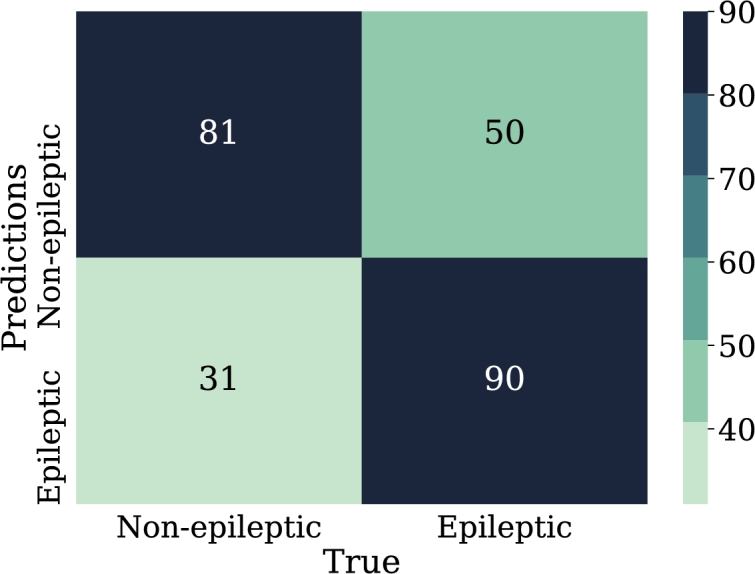
Confusion matrix of model SVM-04. From left to right and from top to bottom the number of true negatives, false positives, false negatives, and true positives.

### 4.5 Convolutional neural networks

Since deep learning throughout has been widely applied in different areas (like in medical applications) [53], an implementation was performed applying a convolutional neural network model with the objective of comparing the results among the three conventional techniques previously applied.

Convolutional neural networks allow the construction of models that use images as inputs by applying filters to them. In this case, as in the previous implementations are used signals in the frequency domain. For these experiments, images were generated for the 21 channels for the 40 epileptic and non-epileptic tests, completing 840 images. In this implementation proposal, in order to be comparable with the previous made, each image corresponds to the frequency spectrum calculated with the FFT. It should be noted that in this way it is not necessary to perform measures on specific frequency bands, therefore, more information of the FFT can be available for the classification process.

Initially, each image was generated with a dimension of (288,432,3) generated in RGB format. However, the first experiments performed presented issues of time processing. After adjusting the model hyper parameters, images dimension was modified to (32,32,1). This allows shorter processing time since the dimensions are smaller than those previously used, however, information may be lost. Given that this CNN application uses the spectrum (frequency) figure calculated via FFT, the input image is in grayscale.

Based on the reported in [54], the architecture design consists of 5 convolutional layers. Regarding these three layers, each one has 32 kernels. The first one has a dimension 3×3, the next one 2×2 and the last one 1×1. Each one has a max-pooling layer of 2×2 associated. These first three layers are followed by two convolutional layers, each with 16 filters of dimension 2×2 and 1×1, which are accompanied by max-pooling layers of 1×1. Then, the model outputs from the convolutional part enter to a flatten layer composed of 700-unit using the activation function “ReLu”. The last layer, which returns the prediction of each sample, uses the activation function “Sigmoid”. Finally, for training process is used the “RMSProp” algorithm.

**Figure 24:**
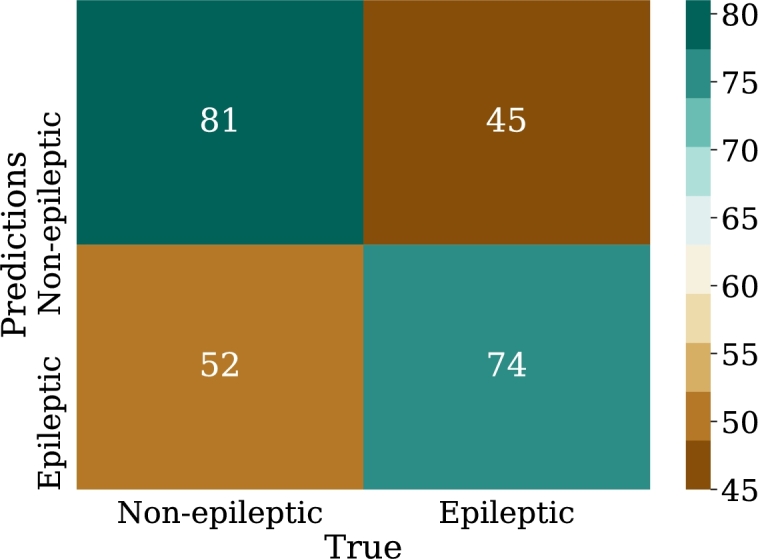
Confusion matrix for the best model of convolutional neural networks implementation. From left to right and from top to bottom the number of true negatives, false positives, false negatives, and true positives.

**Figure 25:**
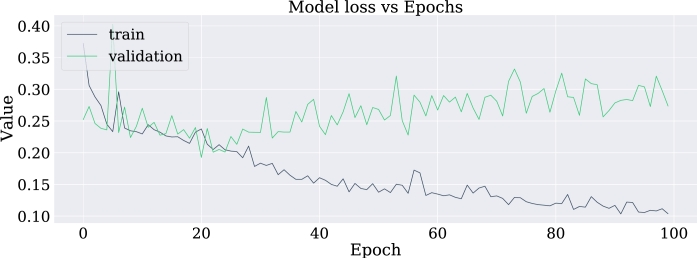
Loss curve of the best model of convolutional neural networks implementation for training and validation data in 100 epoch.

Regarding the results, Fig. 24 shows the confusion matrix for the best model of convolutional neural networks; in addition, Fig. 25 displays the loss curve for training and validation.

## 5 Result analysis

Based on the results obtained in the experimental design, the best two models are obtained. Since each model has three different metrics, the F1-score will be calculated for each model for comparison. This value is the harmonic mean between accuracy and completeness, Equation (10) defines this value.

(10) $$F_{1}=2\times \frac{\text{ precision }\cdot \text{ recall }}{\text{ precision }+\text{ recall }}$$

Table 4, Table 5, Table 6 list the models made in each technique along with their metrics and the calculation of the F1-Score.

**Table 4:** Summary of metrics results for logistic regression models.

Model	Precision	Accuracy	Recall	F1-Score
LOG-REG-01	0.600	0.700	0.720	0.654
LOG-REG-02	0.739	0.733	0.680	0.708
LOG-REG-03	0.673	0.710	0.768	0.717
LOG-REG-04	0.629	0.658	0.702	0.663

**Table 5:** Summary of metrics results for artificial neural networks models.

Model	Precision	Accuracy	Recall	F1-Score
NN-FF-01	0.781	0.765	0.820	0.800
NN-FF-02	0.810	0.861	0.840	0.824
NN-FF-03	0.759	0.742	0.677	0.715
NN-FF-04	0.772	0.805	0.842	0.777

**Table 6:** Summary of metrics results for SVM models.

Model	Precision	Accuracy	Recall	F1-Score
SVM-01	0.796	0.638	0.395	0.527
SVM-02	0.793	0.643	0.387	0.520
SVM-03	0.679	0.702	0.719	0.703
SVM-04	0.775	0.773	0.743	0.758

As seen, the accuracy of the models allows to obtain the fraction of predictions that the model made correctly. This metric is obtained by considering each of the four values contained in the confusion matrix, which at first sight may represent a reasonable calculation for the evaluation of a model. However, with that metric is not achieved a level of detail needed to verify how the model behaves specifically in the classification of epileptic and non-epileptic patients.

Therefore, those metrics are necessary to see the behavior of the model for each class. It is there where the precision and recall metrics appear. With the first one it can see the behavior of the model with respect to the predicted values, and the second metric reveals the behavior with respect to the real values.

Although this seems to solve the issue of model comparison, as seen in Table 5, model NN-FF-01 has better accuracy than model NN-FF-04, which means that the former obtained more real positives within the samples it classified as positive. In spite of this, the model NN-FF-04 obtained a better recall than the model NN-FF-01, that is, the model NN-FF-04 avoids the problem of diagnosing a patient as non-epileptic when in reality is. This same situation can be seen in Table 4 with models LOG-REG-01 and LOG-REG-04. One model presents a better value in one metric but it is worse in another with respect to the other model.

For reasons mentioned above, the F1-score metric is used for the comparison between models since it gathers in itself the precision and recall metrics as can be seen in Equation (10). According to the data in Table 4, Table 5, Table 6, the two best models were NN-FF-01 and NN-FF-02.

Also, a Receiver Operating Characteristic (ROC) graph is used to compare these two models. This curve represents the true positive (TPR) versus false positive (FPR) rate at different classification thresholds. Lowering the classification threshold classifies more items as positive, so both false positives and true positives will increase. Equations (11) and (12) define respectively the values of TPR and FPR.

(11) $$TPR=\frac{TP}{TP+FN}$$

(12) $$FPR=\frac{FP}{FP+TN}$$

Where:

• *TP*: True positives.
• *FP*: False positives.
• *TN*: True negatives.
• *FN*: False negatives.

Even though in this last part strategies have been exposed to evaluate globally the models performance (unify results), there are two metrics based on the values with which the ROC curve is obtained. These are sensitivity and specificity.

• Sensitivity: which measures the proportion of correctly classified epileptic patients [55].
  (13) $$Sensitivity=\frac{TP}{TP+FN}$$
• Specificity: which measures the proportion of non-epileptic patients classified correctly [55].
  (14) $$Specificity=1-FPR=\frac{FP}{FP+TN}$$

**Figure 26:**
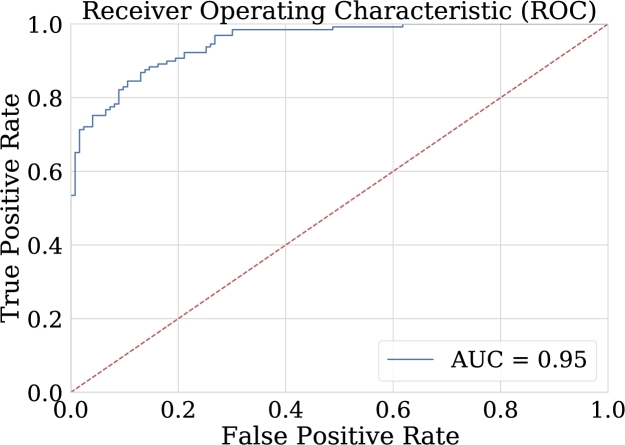
ROC Curve for NN-FF-02 model. This area of 0.95 can be interpreted as the probability that the test classifies correctly when faced with a pair of individuals, one unhealthy and the other healthy.

These metrics illustrate the importance of the ROC curve to visualize model performance for the classification of both epileptic and non-epileptic patients. Therefore, the ideal model will be one in which the FPR is 0 and the TPR is 1. In Fig. 26, it is possible to see how the values on the ROC curve are quite close to these values, different from the graphs of the other two classification techniques [55].

**Figure 27:**
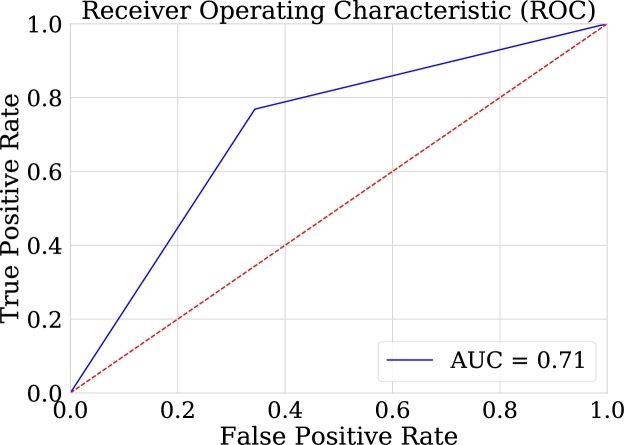
ROC Curve for LOG-REG-03 model.

**Figure 28:**
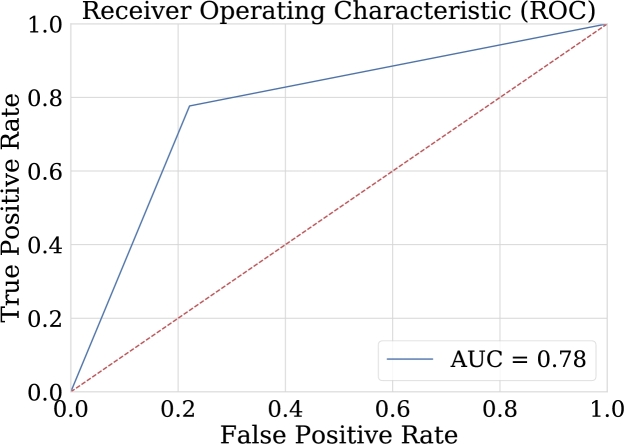
ROC Curve for SVM-04 model.

**Figure 29:**
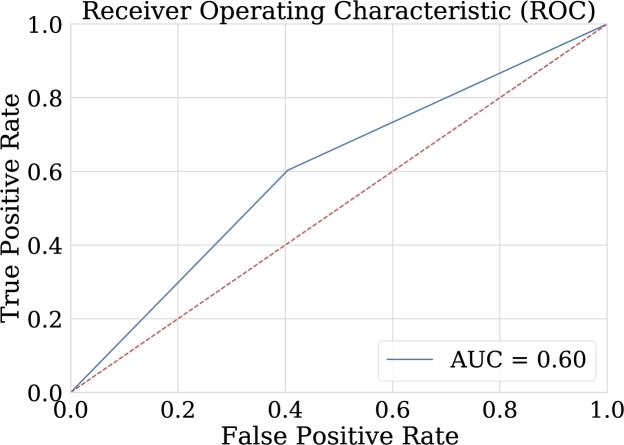
ROC Curve for the best model in convolutional neural networks implementation. This area of 0.60 can be interpreted as the probability that the test classifies correctly when faced with a pair of individuals, one sick and the other healthy.

One advantage of comparing the techniques using the AUC graph is that it allows the visualization of the behavior in the classification. As can be seen in Figure 26, Figure 27, Figure 28, Figure 29 the classification in logistic regression models, support vector machines, and convolutional neural networks, is much lower than in the case of artificial neural networks.

Analyzing the results of the models for LR, ANN, and SVM techniques, it is observed that those models where the data were represented through the average of the frequency bands present better metrics than their opposites, that is, those where the data were represented through the relative power. However, this situation was different for the artificial neural network models since the best results were found when representing the data in relative power. From this, it can be inferred that the preprocessing of the data has a more relevant effect with artificial neural networks and that therefore the separation of the data is easier.

According to Table 7, it can be observed that CNN-01 model performs better than the other models in terms of accuracy, however, the loss is high for this model, despite the capacity of CNN for image classification, it is noted that the best results are not obtained in this case. The reason why these models may have low classification metrics may be that images used does not preserve all the relevant information that distinguishes epileptic patients from non-epileptic patients in the characteristics of the EEG signal. This shows the possibility of carrying out the implementation with another encoding of the figures that are entered to CNN, and other resolution size of the images. In this regard, Fig. 29 shows the ROC curve obtained using CNN.

**Table 7:** Summary of metrics results for CNN models.

Model	Precision	Accuracy	Recall	F1-Score
CNN-01	0.622	0.615	0.587	0.604
CNN-02	0.620	0.611	0.571	0.594
CNN-03	0.606	0.587	0.500	0.547
CNN-04	0.575	0.583	0.635	0.603

Despite the fact that the convolutional neural networks technique is modern and can offer many advantages compared to the traditional techniques used in this study, more explorations than those employed here may be implemented exploring the generation of images from the frequency bands and spectrograms to have more samples that feed the CNN.

## 6 Discussion

The data used in this research was obtained from a public repository at Temple University. This provides a wide state of the art in which these data and the authors' observations regarding the handling and processing of the data were used several times. Although this data source is extensive, it was decided to build a small data set compared to other works, consisting of 20 examinations of epileptic patients and 20 examinations of non-epileptic patients. The addition of more samples may allow better generalization and classification of particular cases.

On the other hand, the application of pre-processing of input data represented improvements in those techniques in which had not been previously applied this strategy. This allowed much more satisfactory results to be obtained for logistic regression, for example, where the classification of the data was not very suitable in the first experiments, also for the CNN model, where modifying the shape of figures represented an improvement on performance and metrics results.

This work focused on using the analysis in frequency and frequency bands to establish the epilepsy that can be used for any channel of the EEG signal. Thus, it is possible to have a classification system with low complexity, giving the possibility of developing a diagnostic device easy to implement. To improve the classification rate, the frequency bands and the channels taken can be used at the same time, which would imply increasing the complexity of the classification system.

In the framework of this research, four of the best known classification techniques were applied; however, this does not mean that they are the four with the best results for this type of problem. Other techniques such as Bayesian classifiers, decision trees or random forest can be applied.

This work is of particular relevance in the area of electroencephalography and machine learning, presenting an approach that allows classifying an exam of a patient through four different techniques. This work could become a starting point for the construction of medical equipment and systems that support neurologists and doctors' work in the diagnosis of epilepsy. It can even be used as alternative or system that acts in those spaces where there is lack of specialists in the field.

## 7 Conclusions

The above was carried out aiming at finding the features and the best classification technique between logistic regression, artificial neural networks, support vector machines, and convolutional neural networks, given the popularity of these techniques in other investigations. Through practical resources and existing models in the Python programming language, it was possible to implement these techniques to the characteristics extracted from the selected EEG exams. A model applying CNN technique was used since it is widely applied among several articles [22]. In this order, this technique presents an alternative to enter the frequency spectrum to the neural network.

In the model training, an improvement in metrics is distinguished from those models in which pre-processing techniques such as scaling and discretization of input data (frequency bands) were applied. An explicit example of such situation is the logistic regression case, in which an improvement of almost 6% in the precision of experiments that did not have feature scaling is observed. This case is exposed given that the logistic regression technique was the one that had rather low metrics and that was not considered satisfactory. For the implementation using CNN, it was convenient convert the images from RGB to grayscale to reduce the computational complexity.

Taking into account the results and the metrics used for comparison in the Results Analysis section, it can be concluded that the most suitable technique for classification of epileptic and non-epileptic patients is the multilayer perceptron ANN technique; particularly, the model NN-FF-02 which displayed the highest precision and accuracy with values 86% and 81% respectively, standing out among all the other experiments. Nevertheless, this does not represent full efficacy in diagnosing patients, it is an outstanding result considering conditions such as size of the data set and the extracted features.

This work is a contribution both in the area of extraction of characteristics of biological signals such as EEG or ECG and also of classification of these signals. These areas are broad and have applications that can be explored to build diagnostic systems whose precision for labeling patients in the Non-epileptic and epileptic classes is superior to what is stated here. A possible extension of the work could be the support and approval by a professional in neurology, who tests the best model of this work with patients whose clinical history and health conditions are varied.

For the expansion of the data set can be considered the addition of others databases; however, it is important the work related to cleaning, normalizing, and removing channels in order to obtain a clean and diverse data set.

In additional work, other classification techniques can be tried as well as other metrics obtained from the EEG signals and configurations of these signals.

### Author contribution statement

M. C. Guerrero, J. S. Parada: Conceived and designed the experiments; Performed the experiments; Analyzed and interpreted the data; Contributed reagents, materials, analysis tools or data; Wrote the paper.

H. E. Espitia: Conceived and designed the experiments; Analyzed and interpreted the data; Contributed reagents, materials, analysis tools or data; Wrote the paper.

### Funding statement

This research did not receive any specific grant from funding agencies in the public, commercial, or not-for-profit sectors.

### Data availability statement

Data included in article/supplementary material/referenced in article.

### Declaration of interests statement

The authors declare no conflict of interest.

### Additional information

No additional information is available for this paper.
